# Therapies for Prevention and Treatment of Alzheimer's Disease

**DOI:** 10.1155/2016/2589276

**Published:** 2016-07-28

**Authors:** J. Mendiola-Precoma, L. C. Berumen, K. Padilla, G. Garcia-Alcocer

**Affiliations:** Laboratorio de Investigación Genética, Facultad de Química, Universidad Autónoma de Querétaro, Cerro de las Campanas S/N, Centro Universitario, 76010 Santiago de Querétaro, QRO, Mexico

## Abstract

Alzheimer's disease (AD) is the most common cause of dementia associated with a progressive neurodegenerative disorder, with a prevalence of 44 million people throughout the world in 2015, and this figure is estimated to double by 2050. This disease is characterized by blood-brain barrier disruption, oxidative stress, mitochondrial impairment, neuroinflammation, and hypometabolism; it is related to amyloid-*β* peptide accumulation and tau hyperphosphorylation as well as a decrease in acetylcholine levels and a reduction of cerebral blood flow. Obesity is a major risk factor for AD, because it induces adipokine dysregulation, which consists of the release of the proinflammatory adipokines and decreased anti-inflammatory adipokines, among other processes. The pharmacological treatments for AD can be divided into two categories: symptomatic treatments such as acetylcholinesterase inhibitors and N-methyl-D-aspartate (NMDA) receptor antagonists and etiology-based treatments such as secretase inhibitors, amyloid binders, and tau therapies. Strategies for prevention of AD through nonpharmacological treatments are associated with lifestyle interventions such as exercise, mental challenges, and socialization as well as caloric restriction and a healthy diet. AD is an important health issue on which all people should be informed so that prevention strategies that minimize the risk of its development may be implemented.

## 1. Introduction

Alzheimer's disease (AD) is an age-related, progressive, and irreversible neurodegenerative disorder characterized by cognitive and memory impairment, and it is the most common cause of dementia in older adults. The estimated prevalence of this disease in 2015 was 44 million people throughout the world and it is estimated that this figure will double by 2050 [1]. Most people with AD (over 95%) have sporadic or late-onset AD (LOAD), a multifactorial disease in which environmental factors and genetic predisposition contribute to the pathology [2]. The other form of AD, familial or early-onset AD (EOAD), corresponds to less than 5% of the AD population and is due to mutations in any of the three following genes: (a) the amyloid precursor protein (APP) gene on chromosome 21, (b) presenilin 1 (PSEN-1) gene on chromosome 14, and (c) presenilin 2 (PSEN-2) gene on chromosome 1 [3–5]. The classification of AD is based on clinical criteria including medical history, physical examination, laboratory tests, neuroimaging, and neuropsychological evaluation [6].

## 2. Pathogenesis and Clinical Features in AD

**Figure 1:**
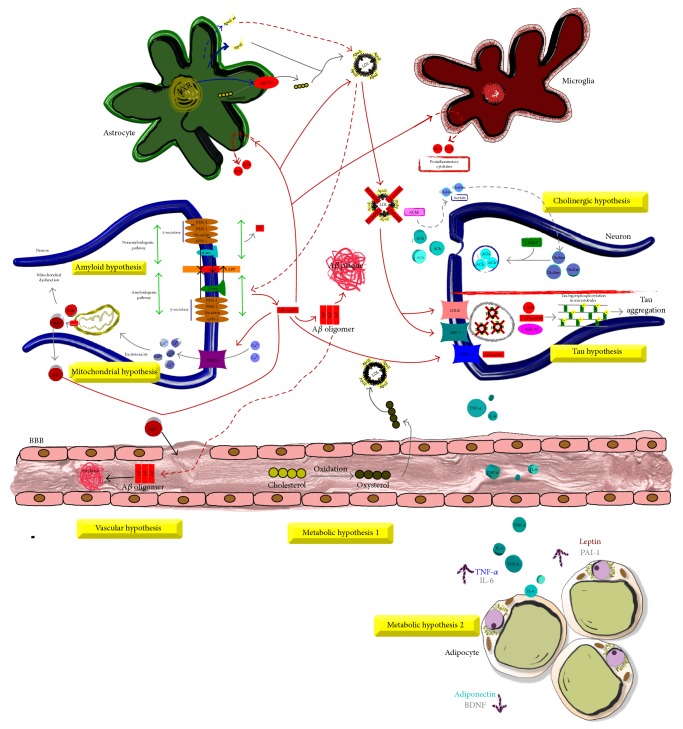
The etiology of AD has been classified in different hypotheses.

The neuropathological features of both forms of AD are characterized by the abnormal extracellular accumulation of amyloid-*β* peptide (A*β*) in amyloid plaques and tau protein aggregated in intracellular neurofibrillary tangles (NFTs). There are epidemiological, clinical, and experimental data that sustain several hypotheses of AD pathogenesis: (1) the amyloid cascade hypothesis proposes that the accumulation of A*β* as neuritic plaques, diffuse plaques, or oligomeric forms in the brain is the main pathogenic event [7]; A*β* plaques are composed primarily of A*β* peptides generated by the amyloidogenic pathway [1]. The amyloidogenic pathway produces amyloid peptides of 39–43 amino acids that are proteolytically derived from the sequential enzymatic action of *β*- and *γ*-secretases on amyloid precursor protein (APP) distributed in the neuron membrane [8, 9] while the nonamyloidogenic pathway produces nontoxic *α*APP fragments that are generated by *α*-secretase action [5]; (2) the tau hypothesis suggests hyperphosphorylation of tau as the primary event [10]; (3) the cholinergic hypothesis proposes that there is a reduction in the activity of choline acetyltransferase and acetylcholine levels in areas such as the cerebral cortex [11]; (4) the mitochondrial cascade hypothesis points to impairment of brain mitochondria as the first pathogenic event leading to neurodegeneration [6]; (5) the metabolic hypothesis holds that the disease is caused by changes in metabolic processes such as obesity, diabetes, and hypercholesterolemia [12]; finally, (6) the vascular hypothesis presents the reduction of cerebral blood flow as the main characteristic [13] (Figure 1).

The clinical diagnosis of AD requires a neuropsychological evaluation according to the criteria of the Diagnostic and Statistical Manual of Mental Disorders (DSM-V criteria) and of the National Institute of Neurological and Communicative Disorders and Stroke and the Alzheimer's Disease and Related Disorders Association (NINCDS/ADRDA criteria). Also, with the progression of the disease, laboratory tests such as oxidative stress products, A*β* levels, oxysterols including 24- and 27-hydroxycholesterol, and proinflammatory cytokines in blood and CSF [6, 7, 14], along with neuroimaging studies such as Magnetic Resonance Imaging (MRI) and Positron Emission Tomography (PET), should be performed [15]. The diagnosis is “probable AD” if cognitive impairment is shown in neuropsychological tests or “possible diagnosis of AD” if there are some positive results of biochemical and neuroimaging tests [2, 16]. It is important to note that, in most cases, but not always, impairment of cognitive domains in which the clinical diagnosis is AD correlates with the neuropathological features of* postmortem* brains with AD [2].

The disease is characterized by pathological changes, including hypometabolism [17], blood-brain barrier (BBB) disruption [13], oxidative stress, mitochondrial impairment [18], and neuroinflammation [19], which can be generated by several metabolic disorders considered strong risk factors for AD. The inflammatory response by activated microglia and astrocytes leading to the production of cytokines and reactive oxygen species (ROS) with associated neuronal damage is another important feature of AD pathogenesis [2].

### 2.1. Risk Factors for LOAD

To minimize the possibility of a future with a high percentage of people with AD, it is necessary to determine which are the factors that influence this disease. In recent years, a significant number of epidemiological studies related to the definition of risk factors for AD have been published. Risk factors for LOAD are classified as susceptibility genes and environmental factors [16]. LOAD has a strong genetic component, namely, apolipoprotein E (ApoE), the most widely studied genetic risk factor for AD. ApoE is produced by the liver, macrophages, and the central nervous system (CNS) [20]. In the CNS, it is produced by astrocytes and microglia; however, neuronal expression of ApoE can be induced in response to stress or neuronal damage under certain pathological conditions (stressors and injurious agents) [21].

The main metabolic and nongenetic risk factors include hypercholesterolemia [22, 23], obesity [24, 25], hyperhomocysteinemia [2], hypertension [26], and type 2 diabetes mellitus (T2DM) [27, 28].

#### 2.1.1. Genetic Susceptibility to LOAD

Apolipoproteins are a family of proteins involved in lipid homeostasis, which bind and transport lipids through the lymphatic and circulatory systems [29]. It has been shown that ApoE has a strong relationship with the pathogenesis of LOAD [21]. ApoE is a glycoprotein of 299 amino acids and its structure varies depending on genetic polymorphisms [30]. The three major ApoE isoforms differ from each other by amino acid substitutions at positions 112 and 158 where the wild-type *ε*3 allele is Cys112 and Arg158, while the ApoE *ε*2 allele carries the Cys112 and Cys158 polymorphism, and the ApoE *ε*4 allele contains Arg112 and Arg158 [31]. A deficiency in ApoE can result in modifications in its structure and function [32], and an alteration of the function of ApoE results in an increase of plasma levels of cholesterol and triglycerides [29].

The ApoE *ε*4 allele is the most important genetic risk factor [29] and it was probably first identified as a risk factor for LOAD, by the initialization and acceleration of A*β* accumulation in the brain [33]. There are numerous studies that have replicated this association in different ethnic groups including African Americans [34], Latinos [35], Asians [36], and Caucasians [37, 38]. One study of the Chinese Han population showed that both the ApoE *ε*4 allele and the CYP17A1 rs743572 allele (a key regulatory enzyme in the steroidogenic pathway) increase the risk of LOAD [36]. Furthermore, a strong association has been reported between the ApoE *ε*4 allele and dementia due to AD pathology, but not with vascular dementia [39]; however, another study produced conflicting results showing that the ApoE *ε*4 allele has a strong relationship with vascular dementia through chronically degenerating white matter in the brain [40]. The mechanisms underlying the association between vascular risk factors and white matter damage are not fully understood.

Genome-wide association studies (GWAS) have identified polymorphisms in several genes that are associated with AD risk, including ABCA7 (which transports substrates across cell membranes), CLU (a stress-activated chaperone protein that functions in apoptosis, complement regulation, lipid transport, membrane protection, and cell-cell interactions), CR1, CD33 (involved in clathrin-independent receptor-mediated endocytosis), CD2AP (implicated in cytoskeletal reorganization and intracellular trafficking), EPHA1, BIN1 (involved in regulating endocytosis and trafficking, immune response, calcium homeostasis, and apoptosis), PICALM (involved in clathrin assembly), and MS4A (associated with the inflammatory response) [41].

#### 2.1.2. Metabolic and Nongenetic Risk Factors for LOAD

*(1) Hypercholesterolemia.* High serum and plasma cholesterol levels have been suggested as risk factors for AD [42, 43]. In the adult brain, primary cholesterol synthesis occurs in astrocytes and in lesser proportion in neurons; cholesterol is transported into the brain by local high density lipoproteins (HDL) [22]. Low-density lipoprotein (LDL) levels are elevated in cardiovascular diseases and increased oxidation and nitration-related systemic modifications are observed in LDL (oxLDL) in hypercholesterolemia [44].

In an experimental cell-based study, cholesterol distribution within membrane is seen to have effects on APP metabolism, trafficking of APP, activities of *β*-, *γ*-, and *α*-secretases, and A*β* synthesis [45, 46]. The mechanism by which cholesterol deregulates A*β* metabolism has not yet been elucidated, but several studies suggest that changes in cholesterol levels alter the cell membrane [44] due to impairment of lipid rafts, which are membrane microdomains focused on protein trafficking [47], signal transduction [48], and neurotransmission [47, 49]. The *γ*-secretase cleavage of APP, the final step in A*β* peptide production, occurs in these cholesterol-rich lipid rafts [44].

In a recent study, it was suggested that inhibition of cholesterol biosynthesis, using AY9944, which blocks the final step of cholesterol biosynthesis, reduces *γ*-secretase activity associated with the generation of A*β* peptides [50]. Moreover, low cholesterol levels increase *α*-secretase activity on APP [51], promoting neuroprotection by increasing levels of *α*APP fragments, which are involved in neurotrophic functions [52]. In another study, it was reported that plasma cholesterol levels in AD patients were elevated by about 10% compared to control subjects [53], though these levels have been linked to the burden of ApoE [54].

The brain is capable of metabolizing cholesterol excess to oxysterols, which are the product of cholesterol oxidation [23]. Several studies have reported some oxysterols including 6-cholesten-5*α*-hydroperoxide, 7-oxocholesterol (7-ketocholesterol), 7*β*-OHC (7*β*-hydroxycholesterol), 7-dehydrocholesterol, 27-OHC (27-hydroxycholesterol), and 25-OHC (25-hydroxycholesterol) [14, 44, 55]. The intermediates 24-OHC and 27-OHC are commonly found in the plasma of patients with AD; thus, these metabolites are very promising as biomarkers in AD patients [56].

*(2) Hyperhomocysteinemia.* Increased levels of homocysteine depend on several factors such as age, genetics, lifestyle, and sex [57]. The causes of this risk factor in the population are multiple and include both nongenetic and genetic mechanisms. Deficiency of vitamin B12, folate, and pyridoxine may be responsible for hyperhomocysteinemia in the general population [58]. Pharmacological data show that homocysteine stimulates lipid accumulation [57], inflammatory processes, and N-methyl-D-aspartate receptor (NMDA) activation [59]. NMDA receptors have been shown to mediate downstream effects of the A*β* peptide in AD models and the pharmacological inhibition of this receptor's activity deletes the pathological effect of A*β* [60, 61].

*(3) Hypertension.* Several studies have linked hypertension to brain atrophy and the generation of NFTs; therefore, an association between hypertension and AD is conceivable [62]. However, this association is complex and differs with age. It has been shown that high blood pressure in middle age is associated with an increased risk of AD [63], while other studies found no association between hypertension in the elderly and dementia [26, 64].

*(4) Obesity.* Obesity is a precursor condition for numerous disorders, including hypercholesterolemia, cardiovascular disease, metabolic syndrome, and type 2 diabetes mellitus (T2DM) [17]. This is due to changes in lifestyle, for example, low levels of physical activity, an unbalanced diet, and overnutrition, leading to inflammatory and oxidative stress processes, altering the metabolic pathways necessary for homeostasis [24].

There are several studies linking obesity to increased cognitive decline and AD risk [12, 65, 66] and to central nervous system inflammation [67, 68] through an increase in proinflammatory cytokines [69]. Studies in both human and animal models suggest that particular dietary constituents may be important in modulating AD risk [70]. For example, a diet rich in fatty acids is associated with obesity and thus with a higher risk of AD [71, 72]. It was recently reported that a high-fat diet causes damage similar to that observed in Alzheimer's pathology, such as potentiation of *β*-secretase processing of APP [73], cognitive impairment [74], and mitochondrial damage associated with insulin resistance [75].

Numerous studies have suggested that obesity in midlife is related to a greater risk of subsequent dementia [76, 77], while in a meta-analysis of longitudinal studies it was reported that obesity in late life is not always associated with AD [66]. In contrast, a recent cohort study reported that midlife obesity (measured as body mass index or BMI) reduces dementia risk [78]. The BMI is an obesity index and some studies have shown an association between BMI and AD, with a significant increase in risk for obese individuals [76]; however, adiposity may be a more important factor and predictor of AD risk than BMI [70].

Adiposity is defined as an increase in total body mass by adipose tissue alterations [79]. Notably, the effect of adiposity on AD incidence has been associated with the consequences of chronic hyperinsulinemia on the blood-brain barrier [80]; thus, it is known that midlife obesity is one of the main factors contributing to the development of type 2 diabetes [79]. It is known that adipose tissue produces regulatory molecules called adipokines, which have autocrine, paracrine, and exocrine effects [77]. Adipokine dysregulation has been correlated with AD, producing changes in proinflammatory adipokines such as an increase in TNF-*α*, interleukin 6 (IL-6), and leptin; a decrease in anti-inflammatory adipokines such as adiponectin; decreased brain derived neurotrophic factor (BDNF); and increased plasminogen activator inhibitor-1 (PAI-1) and angiotensin (AGT) [81, 82]. Adipokines are able to cross the BBB and activate their specific receptors in central nervous system regions such as the hippocampus [81]. The most important adipokines mentioned in the literature with regard to AD are leptin and adiponectin.

Leptin is a 16 kDa adipocyte-derived hormone that is secreted in proportion to the adipose stores, with high circulating plasma levels in obesity [83] resulting in leptin resistance, which boosts tau phosphorylation, in turn increasing Alzheimer pathology [84]. In physiological conditions, leptin generates a reduction of body weight by suppressing appetite or increasing energy balance. Several studies have demonstrated that leptin has beneficial effects through the modulation of memory by regulating both long-term potentiation and synaptic plasticity [79]. In a study of peripheral blood adipokines in patients with AD, it was reported that low leptin levels increase AD risk [85]. However, other studies have reported that high leptin levels are associated with a high risk of dementia [86]. Existing data provide evidence that obesity may interfere with the neuroprotective effect of leptin on the brain, possibly by leptin resistance [87]. Leptin resistance is induced by several defects involving the leptin receptor, in BBB transport, cellular transduction, the induction of feedback inhibitors, and biological systems with changes in cellular networks [84]; overactive leptin receptor signaling results in its intense phosphorylation via the (Janus kinase) JAK-2/STAT-3 pathway, which improves suppression of cytokine signaling 3 (SOCS-3) expression [88]. SOCS-3 is a feedback inhibitor of leptin signaling and has been associated with leptin resistance in obesity. Interestingly, leptin signaling downstream effectors such as adenosine monophosphatase-activated protein kinase (AMPK) and mammalian target of rapamycin (mTOR) regulate the activity of glycogen synthase kinase 3*β* (GSK-3*β*) and BACE1, which are involved in tau phosphorylation and A*β* genesis [12, 89].

In addition to its effects on the AMPK pathway, leptin signaling results in the activation of the energy sensor sirtuin-1 (SIRT1), reducing the acetylation of the p65 subunit of NF-*κ*B and leading to a reduction of tau phosphorylation and of A*β*_1–40_ production [89–91]. Moreover, it has been reported that AMPK and SIRT brain activity is inhibited by prolonged states of positive energy balance, as in obesity [92]. Leptin, as a trophic factor, activates different cascades and, for example, is critical in spine formation in hippocampal neurons; in culture, this is promoted through the phosphorylation of calcium/CaM-dependent kinase *γ* (CaMKI *γ* and *β*-pix), which are required for the trafficking of transient receptor potential-canonical (TrpC1/3) channels to the membrane [93]. Hence, leptin resistance and aging resulting in low levels of this protein are correlated with cognitive impairment and participation by other cytokines.

The adipokine adiponectin is a 30 kDa protein released by adipose tissue and the most abundant adipokine in plasma [94]. Unlike leptin, it is inversely correlated with fat mass; that is, circulating adiponectin levels decrease with increasing adiposity [83]. Adiponectin is a sensor of insulin and can induce body weight loss [95] and insulin resistance, with high levels of tumor necrosis factor (TNF) in plasma and adipose tissue [96]. Adiponectin has been found to modulate some brain functions such as memory and to produce a neuroprotective effect on hippocampal neurons [97, 98]. Furthermore, adiponectin levels are inversely associated with insulin resistance, obesity, type 2 diabetes, and AD. The mechanisms involving adiponectin in the brain are still unclear; however, many epidemiological studies have reported that, for both AD patients and T2DM patients, plasma adiponectin levels are significantly lower than those of healthy individuals [98], although a recent study did not produce the same results [99].

Adiponectin and TNF-*α* inhibit each other's production in adipose tissue, and adiponectin does the same with IL-6, in addition to modulating inflammatory responses, inhibiting the NF-*κ*B-induced pathway [81, 82]. An increase of NF-*κ*B activity in inflammatory status promotes aspartyl protease *β*-site APP-cleaving enzyme (BACE1) synthesis, thus enhancing APP cleavage, and A*β* genesis where low adiponectin levels are associated with AD [82, 100].

The increase in proinflammatory adipokine TNF-*α* levels promotes neuroinflammation as well as inhibition of neuronal proliferation and differentiation [101]. The mechanism involved in these neurotoxic effects is as follows: increase in the production of other cytokines such as IL-6 and leptin, increase of AMPA receptor activity as well as secretases by the nuclear factor of kappa light polypeptide gene enhancer in B-cells inhibitor (I*κ*B) degradation pathway, and overactivation of NF-*κ*B, which may stimulate APP production [81, 102].

The interleukin IL-6, also associated with AD, induces inflammation, inhibition of neurogenesis, and decrease of synaptic plasticity in the hippocampus by triggering neuroinflammation via STAT3, exerting cAMP response element-binding (CREB) protein downregulation by Akt inhibition or by the activation of transcription factors, which compete for a limited pool of coactivators such as STAT-1, c-Jun, and NF-*κ*B, promoting hypocholinergic signaling [103–105].

A member of the neurotrophin family, BDNF, is expressed in adult neurons at high levels throughout the CNS [106] and a decrease thereof has been reported in obesity [107]. Low levels of BDNF have been related to many diseases such as AD because such levels induce a decrease in mitochondrial biogenesis, neuronal survival, and plasticity associated with a deficiency in the TrkB signaling receptor by different pathways: MAPK, a regulator of neuronal differentiation and maturation; PI3K, important in neuronal survival and synaptic protein formation; and PLC*γ*, which induces the release of intracellular Ca^2+^ related to synaptic plasticity [108]. Decreased BDNF also induces apoptosis in neurons by the inhibition of the antiapoptotic family Bcl-2 and the expression of the proapoptotic proteins Bax and Bad, promoting mitochondrial biogenesis [109].

The adipokines related to vascular health are PAI-1 and AGT, which are involved in cell migration and are related to learning and memory process. PAI-1 can be produced by adipocytes, microglia, and astrocytes; the increase of this adipokine in obesity is related to inflammation (an AD risk factor) and fibrinolysis. Moreover, PAI-1 has also been discussed as regards its possible neuroprotective effect associated with the MAPK/ERK pathway [81].

AGT is produced by the liver and white adipocyte tissue in order to increase blood pressure. Increased AGT has been reported in obesity and is considered a risk factor for AD because it increases blood pressure and inflammation [110].

*(5) Type 2 Diabetes Mellitus (T2DM).* Type 2 diabetes mellitus (T2DM) is another prevalent disease associated with obesity and aging, and it is considered an independent risk factor for AD [111]. T2DM and obesity are diseases that affect millions of people worldwide [28, 112]. T2DM is characterized by hyperglycemia resulting in production of increased hepatic glucose, impairment of insulin production by pancreatic *β*-cells, and insulin resistance [27]. Glucose is the only required source of energy for neurons and any disruption in glucose metabolism leads to compromised neuronal functions [113].

The proposed scenarios between diabetes and dementia are numerous; they include vascular lesions, inflammation, oxidative stress, elevated end products of glycolysis, insulin resistance, abnormal insulin receptor signaling, and degradation of insulin and its relation to A*β* protein deposits [114, 115]. Interestingly, both pathologies present amyloidogenesis that forms A*β* plaques [28]. High glucose levels and insulin resistance have a likely impact on oxidative stress pathways and neuroinflammatory signals in the brain, thereby connecting diabetes to neurodegeneration [112]. Furthermore, many researches sustain the hypothesis that AD responds to neuronal pathogenic energy imbalance produced by impairment in the function of glucose [116].

Insulin is a relevant molecule in the regulation of metabolism and energy expenditure. In the brain, insulin is considered a paracrine/autocrine effector, binding to insulin receptors (IRs) and activating the IR substrate (IRS) in two canonical pathways, the phosphoinositide-3 kinase (PI3K)/Akt and the Ras/mitogen-activated kinase cascades [117]. Central insulin is considered to regulate structural and functional aspects of synapses, and neuron-specific insulin receptor knockout (NIRKO) in mice, producing defects in Akt-Foxo3, an insulin signal, develops insulin resistance and causes increased activation of GSK-3*β* and tau hyperphosphorylation [118, 119]. Insulin resistance impairs IR/PI3K/Akt/mTOR insulin signaling, promoting decreased GLUT4, AMPA, and NMDAR exportation to the membrane. Taken together, all these events result in glutamate neurotransmission and long-term potentiation (LTP) dysfunction and tau hyperphosphorylation [117]. It is also important to note that A*β* peptide can bind to the IR and also to ApoE; ApoE itself binds to the IR, and the different interactions between ApoE isoforms suggest that the ApoE *ε*4 genotype could lead to earlier impairment of brain insulin signaling [120].

*(6) Gastrointestinal Microbiota.* Microbiota composition changes have been related as factor risk for several diseases such as obesity, atherosclerosis [121], and T2DM [122] in addition to gastrointestinal disease. More recently, microbiota has been involved with AD due to the possible infectious etiologies of neurogenerative diseases [123]. In this context, previous studies reported associations between* Chlamydophila pneumoniae* [124] as well as herpes simplex virus infection and AD [125]. The possible mechanisms that link microbiota to AD include (1) interactions between the gut microbiota and the CNS in a “microbiota-gut-brain axis” [123], which modify immune response, enhancing response to cerebral A*β* [126], (2) microbiota that could promote prion-like behavior of amyloid proteins leading to neurodegeneration [127], and (3) microbiota changes during aging such as the increase in the proportion of Bacteroidetes to Firmicutes as well as the reduction of bifidobacterial counts [128], which decrease the synthesis of proinflammatory cytokines [129]. Finally, epidemiological links between oral bacteria and AD have been reported [121], due to the increase of TNF-*α* production [130]. Interestingly, oral and gut microbiota can be modified by diet.

## 3. Pharmacological Treatment

Alzheimer's disease requires precise diagnosis, early if possible, and adequate etiological treatment, and, as an incurable age-related neurodegenerative disorder, its particular pathophysiology needs to be considered. The therapeutic options have focused on ameliorating the symptoms as well as reducing the rate of progression of damage, although this has not significantly reversed the disease, so prevention is a better solution for this public health problem [4, 131].

The toxic conformations of A*β* or tau in the brain are thought to spread the disease, and blocking the generation of these peptides may be part of useful treatments. Nevertheless, the current treatments of this disease are based on cholinesterase inhibitors and a glutamate antagonist, providing only symptomatic relief, while evidence for the complexity and multicausality of this dementia is recognized in basic and clinical studies [132]. Efforts in etiology-based treatment are currently underway in clinical trials, as well as complement preventive treatments such as physical activity, proper diet, cognitive stimulation, and the management of comorbidity [133].

### 3.1. Symptomatic Treatment

#### 3.1.1. Acetylcholinesterase Inhibitors

It is well known that acetylcholine (ACh) plays a crucial role in mediating learning and memory [134]. Furthermore, direct interaction between A*β* and cholinergic systems has been proposed, with negative feedback to the production of the peptide; it has been suggested that the alteration in this negative feedback loop and abnormal accumulation of A*β* reduced cholinergic transmission effectiveness, focused on alpha-7 nicotinic acetylcholine receptors [135, 136].

On this basis, effective treatment for AD is achieved with cholinesterase inhibitors, which corresponds well to Davies and Maloney's early cholinergic deficit hypothesis (1976) explaining AD pathophysiology. Tacrine, donepezil, rivastigmine, galantamine, xanthostigmine, para-aminobenzoic acid, coumarin, flavonoid, and pyrroloisoxazole analogs have been developed and studied for the treatment of AD. Rivastigmine, donepezil, and galantamine are the approved drugs that promote higher ACh levels and improve the brain's cholinergic function by inhibiting the enzyme acetylcholinesterase which degrades the neurotransmitter [137–139]. In general, acetylcholinesterase inhibitors (except tacrine) are well tolerated and adverse effects are dose-related [4]. The acetylcholinesterase inhibitor ladostigil (TV3326) is in phase II clinical trials and it also produces antidepressant effects for the inhibition of monoamine oxidases A and B [140].

#### 3.1.2. N-Methyl-D-aspartate Receptor (NMDA) Antagonist

Glutamate-mediated excitotoxicity is known to result in calcium overload and mitochondrial dysfunction, with increased nitric oxide generation, which can be detrimental to cells, forming high levels of oxidants and eliciting neuronal apoptosis. This overstimulation can be blocked by NMDA receptor antagonists such as memantine, which was approved in 2003 by the Food and Drug Administration (FDA) for the treatment of moderate-to-severe AD, with a marginal beneficial effect on cognition in mild-to-moderate AD [4, 141, 142].

Memantine can protect neurons by attenuating tau phosphorylation through a decrease in glycogen synthase kinase 3*β* (GSK-3*β*) activity. This noncompetitive glutamatergic NMDA receptor antagonist can be administered alone or in combination with an acetylcholinesterase inhibitor [143], although there may be few significant favorable changes in the combination therapy [137].

#### 3.1.3. Other Neurotransmitter Systems

Muscarinic and nicotinic ACh receptors are also considered targets for AD treatment, although selectivity of the agonists has been a problem outcome in clinical trials. EVP-6124 is currently in phase II trial [131].

Based on the cholinergic hypothesis and NMDA glutamate participation in AD, it is natural to consider the different neurotransmitter networks, particularly of the hippocampus. Serotonin receptors are expressed in areas of the CNS involved in learning and memory. The inhibition of 5-HT_6_ serotonin receptors was shown to promote acetylcholine release, and some compounds are in various stages of clinical research, considered as possible treatments for mild-to-moderate AD [140].

Histamine receptors, particularly H_3_ receptors, are also present in large amounts in memory- and cognition-related structures in the brain. It seems that H_3_ receptor antagonists may improve cholinergic neurotransmission. Phase I and II studies with H_3_ antagonists are currently being conducted [139].

### 3.2. Etiology-Based Treatment

As indicated above, ApoE *ε*4 is the major genetic risk factor for sporadic AD (the major risk factor is age), although, for disease-modifying treatment based on the amyloid cascade hypothesis, efforts are targeting secretase modulation and amyloid binders, as well as targeting kinases involved in the hyperphosphorylation of tau protein [131, 132, 140].

#### 3.2.1. Secretase Inhibitors

APP is first cleaved either by *α*-secretase or by *β*-secretase enzymes, and the resulting fragments are processed by *γ*-secretase. The proposal of the “overactivation” of *β*- and *γ*-secretases, or age-related decreased *α*-secretase processing, has led to the use of inhibitors for this amyloidogenic pathway [144].

Several metalloproteinases have been studied with *α*-secretase activity. The upregulation with gemfibrozil (PPAR-*α* agonist) of the *α*-secretase “A disintegrin and metalloproteinase” 10 (ADAM10) has been proposed as a good strategy for the prevention of A*β* generation [145]; melatonin also stimulates the nonamyloidogenic processing of APP through positive transcriptional regulation of ADAM10 and ADAM17 [146] and stimulation with serotonin 5-HT_4_ receptor agonists regulates *α*-secretase activity [147]. Overexpression of matrix metalloproteinase 9 (MMP-9, another *α*-secretase) also prevents cognitive deficits displayed by the transgenic AD mouse model harboring five familial AD-related mutations (5xFAD) [148].

The transmembrane aspartyl protease BACE1 has inhibitors proposed with a molecular docking-based approach for the inaccessible catalytic center that initially led to unsuccessful trials [131, 149, 150]. BACE1 plays an important role in the metabolism of myelination proteins; however, its inhibition displays less severe side effects than other ADAM proteases. Few compounds have reached clinical trials, the most promising being Merck Sharp & Dohme's MK-8931 (Verubecestat) and Eli Lilly/Astra-Zeneca's AZD3293 (LY3314814), in phase II/III trials NCT01739348 and NCT02245737, respectively [139, 140]. Flavonols and flavones, especially myricetin and quercetin, have exhibited very good cell-free BACE1 inhibitory effects [131].

*γ*-secretase is a transmembrane multisubunit protease complex, composed of presenilin 1, nicastrin, anterior pharynx defective-1 (APH-1), and presenilin 1 enhancer-2 (PEN-2), and it is involved in the proteolysis of many intramembranous signaling proteins. There have been many studies with *γ*-secretase inhibitors, which induced significant side effects, including gastrointestinal disorders and increased risk of skin cancer. One of its substrates is Notch protein, which regulates cell proliferation, differentiation, and growth; *γ*-secretase Notch-sparing inhibitors were designed, although results in clinical trials are not very promising [139]. It seems that *α*-secretase activity is needed to prevent A*β* peptide formation and its age-related downregulation may be compensated by dietary changes including several antioxidants that activate the promoter of the ADAM proteases involved; *γ*-secretase activity is also needed before *β*-secretase reaches APP in order to prevent A*β*, since evidence for genetic defects in *γ*-secretase (PSEN-1 and PSEN-2) as major risk factors for familial AD is conclusive. This may explain why the use of *γ*-secretase inhibitors has failed in early trials but modulators of this complex have better expectations.

The toxicity of *γ*-secretase inhibitors depends on other signaling pathways activated by other cleaved receptors, including Notch receptor [150]. It has been shown that a *γ*-secretase inhibitor, but not a *γ*-secretase modulator, induces defects in BDNF axonal trafficking and signaling [151]. These modulators have effects on the A*β* cleaving site generator without affecting other cleaving sites of the complex [152, 153].

Hypercholesterolemia is considered a risk factor, as stated above, and also the participation of cholesterol in secretase activity has been discussed, but it is more important to recall that many acidic steroids are *γ*-secretase modulators that selectively decrease A*β*42; cholestenoic acid, a cholesterol metabolite, is one of these endogenous modulators [154]; the regulation of these endogenous metabolites may be involved in obesity-induced AD (including other risk factors such as dyslipidemia and metabolic syndrome).

#### 3.2.2. Amyloid Binders

The deposition of A*β* in AD is concentration-dependent; increased amyloidogenic processing of APP and inefficient removal of peptides may be involved in the pathology. There is reduced activity of A*β*-degrading enzymes, such as neprilysin, an insulin-degrading enzyme, as well as the ApoE determinant, which correlates well with the proposal of AD as a metabolic disorder [140].

Preventing the formation of A*β* extracellular neuritic (senile) plaques is one of the targets for disease-modifying treatment in AD, although there is evidence of correlation with A*β* biomarkers and cognitive deficits, previous to senile plaques. Inhibitors of A*β* aggregation have reached clinical trials [140]. In addition, amyloid-*β*-directed immunotherapy includes several biological products involving probable sequestration of soluble monomeric A*β* (solanezumab) or microglia-mediated clearance (bapineuzumab, crenezumab, gantenerumab, aducanumab, and BAN2401) currently in clinical trials [132]. However, active and passive immunization may involve side effects with neuroinflammation, which is considered in itself to explain the pathophysiology of AD, and anti-inflammatory agents for treatment of AD might be considered as well.

#### 3.2.3. Anti-A*β* Aggregation Compounds

In recent decades, research has focused on developing therapies in which the A*β* peptide formation or its aggregation is prevented. Among the small molecule inhibitors of A*β* aggregation in clinical trials are tramiprosate (phase III), clioquinol (phase II), scylloinositol (phase II), and epigallocatechin-3-gallate (phase II/III); although these drugs have achieved stabilization of the A*β* monomers, they have important side effects [155]. Also, synthetic *β*-sheet breaker peptides of the iA*β*5p sequence such as azetidine-2-carboxylic acid, 3-phenyl azetidine-2-carboxylic acid, *β*-proline, and *β*-sulfonylproline modulate the cell damage caused by the A*β* exposure by preventing fibril formation and they have shown improved results with regard to spatial memory [156, 157]. Stemazole has been shown to protect SH-SY5Y cells from toxicity induced by A*β in vitro*, reducing A*β* aggregation [158]. Likewise, compounds such as curcumin, T718MA, and SK-PC-B70M protect neurons from A*β*-induced toxicity [156].

#### 3.2.4. Tau Therapies

Prevention of aggregates of paired, helically twisted filaments of hyperphosphorylated tau in neurofibrillary tangles is one of the targets of this therapy. Immunotherapy has been developed; AADvac1 was the first vaccine in clinical trials, and ACI-35 (another liposomal-based vaccine) trials have begun [139].

Inhibitors of the phosphorylation of tau proteins such as tideglusib, an irreversible GSK-3*β* inhibitor, have been tested with no statistically significant benefits [139]; cyclin-dependent kinase 5 (CDK5), which is also involved in the hyperphosphorylation of tau proteins, has been considered as a possible drug target [140].

Several molecules have been shown to act as good inhibitors of tau aggregation and are in clinical trials. Among these drugs, methylene blue (MB) and its metabolites azure A and azure B are able to promote protein degradation and inhibit caspase-1 and caspase-3 activity [159]. Similarly, leucomethylthioninium with a suitable counterion (LMTX in phase III clinical trials) and methylthioninium chloride or MTC (phase II clinical trial) have been shown to reduce tau aggregation and reverse behavioral deficits in transgenic mouse models [160] and to slow the progression of the disease in patients with AD [10]. However, the exact mechanisms by which LMTX and MTC generate neuroprotective effects* in vivo* are not completely understood. Other promising inhibitors of tau aggregation are N-phenylamines, anthraquinones, phenylthiazolyl-hydrazides, rhodanines, benzothiazoles, and phenothiazines [161].

#### 3.2.5. Other Therapies

As an age-related pathology, AD is correlated with other chronic-degenerative disorders, and coordinated therapies are needed. A type 3 diabetes hypothesis of AD has been developed, and intranasal insulin is included as a possible treatment for the disease, due to its ability to penetrate the brain-blood barrier [139].

Elevated low-density lipoprotein (LDL) concentration increases the risk of developing AD but the use of statins as a protective treatment is controversial [162, 163]. Dyslipidemia and obesity are considered causative factors in relation to other pathologies such as metabolic syndrome, which includes atherogenic dyslipidemia and central obesity, hyperglycemia and insulin resistance, hypertension, and a prothrombotic state and a proinflammatory state [164, 165]. Statins may prevent dementia due to their role in cholesterol reduction, although there is evidence that statins given in late life to people with a risk of vascular disease do not prevent cognitive decline or dementia [166]. There is a reduction in cholesterol levels in the diabetic brain, as well as in neuron-derived cholesterol content, which affects receptor signaling [167], so the use of statins in AD treatment should be with consideration to the early management of the disease.

In addition, drugs used in the treatment of type II diabetes mellitus may have a neuroprotective effect in AD. Amylin and glucagon-like peptide-1 receptor agonist are also under study as AD treatments [139].

Finally, the mitochondrial cascade hypothesis includes oxidative stress, a state of lost balance with overproduction of oxidative free radicals as well as reactive oxygen species (ROS) and reactive nitrogen species (RNS) [141, 168]. This imbalance also includes the participation of immune cells and NO signaling, and preventive treatment with antioxidants (see Nonpharmacological Treatments) and anti-inflammatory drugs is considered [169]. What is certain is that prevention is our best strategy for AD, with efforts to prevent obesity and chronic-degenerative disorders.

## 4. Nonpharmacological Treatments

Nonpharmacological treatments are important for the prevention of AD or as adjuvants in other treatments. AD prevention strategies can be divided into two groups, the first associated with lifestyle and the second with diet and chemical compounds.

### 4.1. Lifestyle

Lifestyle strategies include physical activity, mental challenges, energy restriction, and socialization as preventive factors in AD [170]. Physical activity such as aerobic exercise was associated with the reduction of AD deficits in a cohort study [171]. This was not consistent with studies that considered a small number of cases [172].

Exercise was reported to enhance hippocampal neurogenesis [173, 174] and learning in aging rodents [175]. The three mechanisms proposed in order to explain the exercise neuroprotective effect of exercise are (1) the release of neurotrophic factors such as BDNF and insulin-like growth factor (IGF-1), nerve growth factor (NGF), and vascular endothelial growth factor (VEGF) [138, 176] from neurons in synaptic activity, which stimulates neurogenesis and synaptic neural plasticity through the stimulation of CREB transcription factor; (2) the reduction of free radicals in the hippocampus as well as the increase in superoxide dismutase and endothelial nitric oxide synthase [176]; (3) peripheral signals that help to support the demands of active neuronal networks such as BDNF release in addition to energy restriction on the brain [109, 177–179].

It has been proposed that mental challenges may protect against cognitive decline and probably against AD [180]. Computer courses and psychoeducation have moderate beneficial effects [181]. Stimulation by cognitive activities has been associated with an increase in neuronal density, which provides brain reserve and plasticity [170].

The relation between caloric restriction and brain motivation is important since many years ago humans needed to obtain their food by killing wild animals and often vigorous exercise was required [182]. In different AD mouse models treated with food and caloric restriction, a decrease in phosphorylated tau and amyloid-*β* was observed in the brain. The possible mechanism may be associated with SIRT1, a protein with nicotinamide adenine dinucleotide-dependent deacetylase or adenosine diphosphate-ribosyltransferase activity [183], because its increase was reported in p25 CK mice with characteristics similar to AD. In addition, SIRT I stimulation by resveratrol induces neuronal death protection. SIRT1 levels also increase with NADp* in vitro*, and SIRT induces an increase of *α*-secretase and a decrease of *β* amyloid deposition in primary cultures in a mouse model of AD [184]. The relationship between hunger and neuroprotection was induced by ghrelin in a mouse model of AD; the results indicated improved cognition in the water maze test and a decrease in amyloid-*β* levels and inflammation [185].

Socialization is important to mental and physical human development and a lack thereof induces loneliness, which has been associated with various diseases such as depression, alcohol abuse, obesity, diabetes, hypertension, AD, and cancer [186].

### 4.2. Diet and Chemical Substances

Dietary supplements for prevention of AD were studied with vitamins such as B6, B12, folates, and E, C, and D vitamins. Vitamin B studies produced mixed results; on one hand, a two-year treatment with homocysteine and vitamin B in 271 patients indicated a significant difference compared to placebo in whole brain atrophy [187, 188], whereas other reports indicate different results [189, 190]. It has been proposed that folic acid has neuroprotective activity through an epigenetic mechanism that inhibits amyloid-*β* peptide accumulation. Studies with 2000 IU of vitamin E did not indicate a protective effect for AD with three years of treatment [191], nor with the combined treatment with vitamin C [192]. Additionally, vitamin D supplementation improves cognitive performance [193].

With regard to the intake of chemical substances, the results in alcohol studies indicate an association between the prevention of AD and low levels of red wine consumption [194] due to its polyphenols composition, whereas drinking alcohol frequently was associated with a risk of dementia [195]. Different molecules have been proposed for their neuroprotective effect, including glucosamine, omegas 3 and 6 which induce interleukins or prostaglandins for inflammatory responses [196], and antioxidants such as *β*-carotene and lycopene 6 [197].

Other studies of chemical substances related to possible protection against neuropsychiatric disorders such as AD were those related to the intake of plants and their secondary metabolites: flavonoids, alkaloids, or terpenoids [198, 199]. Flavonoids are considered safe [200] and their neuroprotection was confirmed in 90 people treated with flavanol [201]. Flavonoids also inhibit acetylcholinesterase and improve memory [202], in addition to inhibiting glutamate release [203].

Resveratrol is a polyphenol found in various plants, especially berries, peanuts, and red grapes, as well as in red wine [204]. This polyphenol has shown various biological activities such as antioxidant, anti-inflammatory, phytoestrogen, vasodilator, cardioprotective, and anticarcinogenic activities, while many studies have proposed resveratrol as a molecule with therapeutic potential in neurodegenerative diseases such as AD [205]. Neuroprotective functions of resveratrol in the pathogenesis of AD have been evaluated through different mechanisms of action. The neuroprotective effects of resveratrol have been associated with the modulation of transcription factors NF-*κ*B, cAMP, p53, and cyclins, as well as an increase in BDNF among others related to mitochondrial biogenesis, oxidation of fatty acids, and suppression of proinflammatory molecules [206].

One mechanism by which resveratrol generates anti-inflammatory effects is by proinflammatory cytokine activity inhibition (IL-1*β*, IL-6, and TNF-*α*) and prostanoid synthesis, principally prostaglandin E2 (PGE2) [207]. Furthermore, it has been shown that resveratrol has the ability to activate sirtuin, particularly SIRT1, leading to the protection of neurons from apoptotic processes and oxidative stress [208]; it has also been demonstrated that activation of SIRT1 induced by resveratrol reduced NF-*κ*B signaling pathway activation in glial cells exposed to A*β* [209]. Under normal cellular conditions, SIRT1 is activated by AMPK, and then activated SIRT1 is deacetylated to transcription factors such as peroxisome proliferator-activated receptor gamma coactivator 1-alpha (PGC1-*α*), which translocates to the nucleus and interacts with peroxisome proliferator-activated receptor (PPAR-*γ*) to enhance gene expression, which promotes cell survival and the proper functioning of mitochondria [206, 210].

Another flavonoid is luteolin which has been reported to exhibit significant action in AD prevention associated with its antioxidant, anti-inflammatory, and microglia-inhibiting effects along with improved spatial memory [211]. Luteolin also inhibits multiple transduction signals such as NF-*κ*B, PKC, STAT3, and intracellular calcium [212, 213].

The Mediterranean diet may improve neuroprotection because it is based on low intake of saturated fatty acids, but high consumption of unsaturated fatty acids, as well as vegetables, legumes, fruits, fish, and olive oil, along with polyphenols such as oleuropein aglycone (OLE), which interfere with amyloid aggregation, and reduced the LDL cholesterol levels. The monosaturated fatty acids have been reported with antioxidant and anti-inflammatory effects, as well as endothelial function improvement and less cognitive decline, whereas polysaturated fatty acids are important in neuronal membrane integrity and function; omega 3 was related to gene expression that might influence the inflammatory process, nerve membranes neuroplasticity, and synaptic transmission [214–218]. Another diet related to neuroprotection against neurodegenerative diseases is the Asiatic diet, because it includes high levels of green tea consumption, the antioxidant curcumin, and the dietary supplement* Gingko biloba*, considered to be a protector against memory decline, due to its antioxidant effect and the decrease of A*β* aggregation; it is necessary to increase the research in order to know its toxic effects [138]. On the other hand, the western diet is considered as a risk factor for AD because it is characterized by excessive consumption of sugar and animal products, with a higher content of saturated fats, which negatively affect cognitive function, A*β*-deposition, and oxidative stress [218].

OLE is an important compound with neuroprotective effects because it interferes with amylin, tau, and A*β* peptide aggregation and toxicity* in vitro*, studied by behavioral, biological, biophysical, biochemical, and electrophysiological techniques. OLE also has pharmacological activities such as cardioprotective, antioxidant, anticancer, antimicrobial, and antiviral effects; this compound also prevents low-density lipoprotein oxidation and platelet aggregation, inhibiting eicosanoid production, and it produces an effect against metabolic syndrome: obesity and type 2 diabetes and hepatic steatosis induced by a high-fat diet in mice, probably associated with WNT expression downregulation of the inhibitor genes and toll-like receptor and of the proinflammatory cytokines. OLE also downregulates several transcription factors and their target genes involved in adipogenesis and upregulation genes such as *β*-catenin [214].

**Figure 2:**
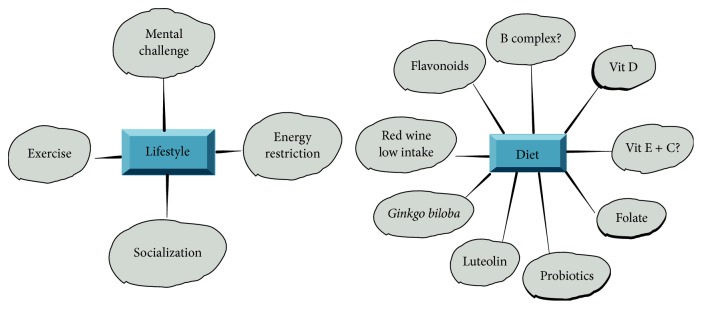
Nonpharmacological treatments.

Another nonpharmacological treatment could be the intake of probiotics due to their reduction of the proinflammatory cytokines associated with gut microbiota changes during aging [128] (Figure 2). Probiotics administration in the elderly may improve gut health and boost anti-inflammatory activity [219], since the “microbiota-gut-brain axis” can decrease the neuroinflammatory process. Furthermore, the beneficial effects of probiotics in AD have been associated with their production of metabolites by fermentation, including short-chain fatty acids (SCFAs) such as propionic and butyric acids [220]. A recent study reported a neuroprotective effect of* Clostridium butyricum* which restored brain levels of butyrate in a mouse model of vascular dementia [221]. Probiotics increase intestinal barrier integrity by activating epithelial cells protecting against pathogens [222]. In addition, previous work showed downregulation of TNF-*α* levels and an increase in IL-10 production resulting from the administration of* Lactobacillus rhamnosus* [223]. It is important to note that the intake of probiotics, such as* Lactobacillus plantarum*, may also induce behavioral changes, through monoamine neurotransmitter augmentation [224].

AD is a multifactorial disease and the combination of two or more nonpharmacological treatments for prevention is important, in addition to pharmacological treatments.

AD should be considered at early ages in order to avoid risk factors, and, for elderly individuals, increasing treatment to a combination of two or more nonpharmacological treatments strengthens prevention. In cases where dementia is present, it is important to improve treatments by adding lifestyle and dietary changes. AD research needs to be further developed in order to propose new molecules for therapy and prevention.
